# Sinew acupuncture for knee osteoarthritis: study protocol for a randomized sham-controlled trial

**DOI:** 10.1186/s12906-018-2195-8

**Published:** 2018-04-23

**Authors:** Kwok Yin Au, Haiyong Chen, Wing Chung Lam, Chiu On Chong, Andrew Lau, Varut Vardhanabhuti, Kin Cheung Mak, Fei Jiang, Wing Yi Lam, Fung Man Wu, Hiu Ngok Chan, Yan Wah Ng, Bacon Fung-Leung Ng, Eric Tat-Chi Ziea, Lixing Lao

**Affiliations:** 1 Hong Kong Institute of Integrative Medicine, Faculty of Medicine, The Chinese University of Hong Kong, Hong Kong, China; 2 School of Chinese Medicine, The University of Hong Kong, 10 Sassoon Road, Pokfulam, Hong Kong, China; 3 Department of Chinese Medicine, The University of Hong Kong-Shenzhen Hospital, Shenzhen, China; 4 The Hong Kong Tuberculosis Association Chinese Medicine Clinic cum Training Centre of the University of Hong Kong, Hong Kong, China; 5 Department of Diagnostic Radiology, Li Ka Shing Faculty of Medicine, The University of Hong Kong, Hong Kong, China; 6 Department of Orthopaedics and Traumatology, Li Ka Shing Faculty of Medicine, The University of Hong Kong, Hong Kong, China; 7 Department of Statistics and Actuarial Science, The University of Hong Kong, Hong Kong, China; 8 The Chinese Medicine Department, Hospital Authority, Hong Kong, China

**Keywords:** Sinew acupuncture, Sham acupuncture, Knee osteoarthritis, Pain, Randomized controlled trial, Protocol

## Abstract

**Background:**

Sinew acupuncture is a new modality of acupuncture in which needles are inserted into acupoints, ashi points or spasm points of sinew and muscles along the meridian sinew pathway. A previous observational study revealed that sinew acupuncture has immediate analgesic effects on various soft tissue injuries, including knee injuries. However, no rigorous trials have been conducted. This study aims to examine whether sinew acupuncture can safely relieve pain and symptoms of knee osteoarthritis (KOA) and improve patients’ functional movement and quality of life.

**Methods/design:**

A randomized, sham-controlled, patient- and assessor-blinded trial will be conducted to compare the efficacy of sinew acupuncture and sham acupuncture. Subjects will be assessed by the physician and acupuncturists. A sample of eighty-six eligible subjects will be randomized into either the sinew acupuncture group or the sham acupuncture group. The intervention will be performed in the Hong Kong Tuberculosis Association Chinese Medicine Clinic cum Training Centre of the University of Hong Kong by acupuncturists with over 3 years of acupuncture experience. Subjects will receive 10 sessions of interventions for 4 weeks, followed by a 6-week follow-up. The visual analogue scale (VAS) score at week 4 will be the primary outcome. The Western Ontario and McMasters University Osteoarthritis Index (WOMAC), Timed Up & Go Test (TUG), 8-step Stair Climb Test (SCT) and the 36-Item Short Form Survey (SF-36) will be secondary outcomes.

**Discussion:**

Sinew acupuncture is a potential alternative non-pharmacological therapy for KOA. This rigorous trial will expand our knowledge of whether sinew acupuncture reduces pain intensity and improves symptoms, functional movements, and quality of life of KOA patients.

**Trial registration:**

The study was registered at ClinicalTrials.gov (Identifier: NCT03099317) in March 2017.

## Background

Osteoarthritis is one of the leading causes of disability in the elderly worldwide [1, 2]. The knee is the most commonly affected site [3]. Hip and knee osteoarthritis was ranked the 11th highest contributor to years lived with disability globally [4]. A US population-based survey found that 30.8% of men above the age of 65 and 34.8% of elderly women have radiographic evidence of knee osteoarthritis (KOA) [5, 6]. A survey study with the same protocol conducted in China found that the prevalence of KOA in elderly men (27.6%) was similar, but the prevalence in elderly women (46.6%) was higher than that in the US study [6]. Furthermore, the two studies revealed that the prevalence of pain in KOA was 6.9% to 7.1% in elderly men and 11.6% to 15.4% in elderly women [6]. In 1997, the cost of osteoarthritis in the USA, Canada, UK, France, and Australia accounted for up to 1–2.5% of the gross national product in each of these countries [7]. Approximately one-third of direct osteoarthritis expenditures were from medications, mainly for pain-related agents [8]. The burden of osteoarthritis will continue to increase worldwide in the ageing population.

Knee pain largely affects KOA patients’ quality of life and is the major reason patients seek medical help and advice [9]. Many modalities of non-pharmacological, pharmacological and surgical therapies have been implemented to reduce pain in KOA patients, including acupuncture, exercise, non-steroidal anti-inflammatory drugs (NSAIDs), COX − 2 selective agents, and joint replacement surgery [10]. NSAIDs and COX-2 inhibitors are commonly prescribed medications [10] but are associated with an increased risk of gastrointestinal bleeding and cardiovascular events [10].

Acupuncture has been widely used for KOA, although large-scale randomized controlled trials have shown contradictory results [11–14]. The discrepancies are caused by various factors such as the characteristics of controls [15, 16] and the dose of acupuncture treatment [16]. Systematic reviews have indicated that acupuncture is beneficial for patients with KOA [17, 18] and that the effects of acupuncture last for over 12 months [19].

Sinew acupuncture, a specific modality of acupuncture, was named by Professor Nongyu Liu. It was developed following the principles of *Huangdi Neijing*, a classic Chinese medical text, in conjunction with clinical experience [20–22]. *Huangdi Neijing* means “taking the [points of] tenderness as acupoints”. Needles are inserted superficially at sinew points (spasm points, painful points, or acupoints close to or distal to the pain) along the meridian sinew pathway to achieve therapeutic effects [22, 23]. TCM treatments, based on the theory of meridian sinews, have recently been used for pain management [24–27]. Sinew acupuncture potentially reduces the risk of internal organ injuries. Sinew acupuncture generally causes less pain than traditional acupuncture, as it does not require deep insertion of needles or the Deqi sensation by manual manipulation of the needle, such as lifting, thrusting, twisting, and twirling [28].

Our previous observational studies have indicated that sinew acupuncture has immediate analgesic effects on soft tissue injuries at various locations (knee, elbow, back, neck and shoulders) [29]. As no controls were used in the previous observational studies, it is unclear whether the immediate analgesic effects are due to true effects or spontaneous remission of the tissue injury.

The proposed study aims to examine whether sinew acupuncture can relieve pain and symptoms of KOA and improve functional movement as measured by the visual analogue scale (VAS), the Western Ontario and McMasters University Osteoarthritis Index (WOMAC), the Timed Up & Go Test (TUG) and the 8-Step Stair Climb Test (SCT). We will also evaluate whether sinew acupuncture can improve quality of life (QOL) as measured by the 36-Item Short Form Survey (SF-36). Furthermore, we will evaluate the safety of sinew acupuncture in treating KOA. We hypothesized that sinew acupuncture can safely relieve pain, enhance functional movement and improve QOL in KOA patients compared to sham acupuncture.

## Methods/design

### Study design

**Fig. 1:**
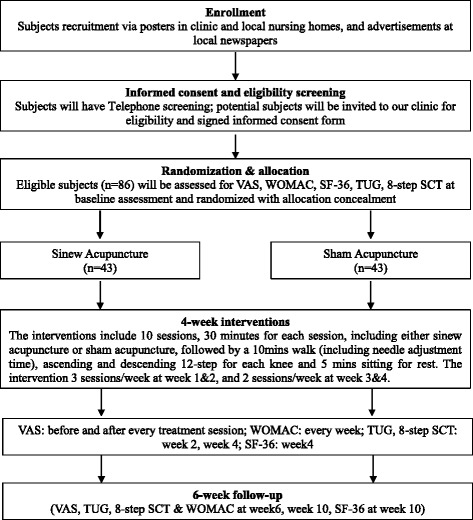
Flow diagram of the study protocol. Assessment measures will be performed as follows: (1) Pain: VAS (visual analogue scale) and WOMAC (Western Ontario & McMasters University Osteoarthritis Index); (2) Functional movement: TUG (Timed up & Go Test) and 8-step SCT (Stair Climb Test); (3) Quality of life: Short Form-36 (SF-36); (4) Credibility test (end of weeks 2 & 4); (5) Test of blinding success (end of week 4); (6) Patient diary (weekly)

A randomized, sham-controlled, patient- and assessor-blinded trial will be conducted to compare the efficacy of sinew acupuncture and sham acupuncture. Subjects will receive 10 sessions of interventions, either sinew acupuncture or sham acupuncture, for 4 weeks followed by a 6-week follow-up period. The interventions will be performed by acupuncturists with more than 3 years of experience in acupuncture practice. Subjects will be blinded to the intervention and assessed by independent assessors using the primary outcome measure, VAS, and the secondary outcome measures, WOMAC, TUG and 8-step SCT. The details of the study design are shown in Fig. 1.

### Study subjects

Subjects will be recruited from the Hong Kong Tuberculosis Association Chinese Medicine Clinic cum Training Centre of the University of Hong Kong, local nursing homes and community centres using advertisements in local newspapers. The eligibility of subjects will be assessed by the physician and acupuncturists using the criteria described below.

### Inclusion criteria

Subjects are eligible to participate if they meet the following criteria: (i) are male or female Hong Kong permanent residents aged 50 years or above; (ii) meet the Clinical Classification Criteria for Osteoarthritis of the Knee as recommended by the American College of Rheumatology, have knee pain, have radiologic findings of osteoarthritis with Kellgren and Lawrence Grades 2–4, and have less than 30 min of morning stiffness or crepitus on active motion and osteophytes as determined by history and physical examination; (iii) have either unilateral knee pain or bilateral knee pain; (iv) have experienced pain for at least 6 months and knee pain > 40 mm on a visual analogue scale (VAS; 0 to 100 mm) within the past 7 days; and (v) are able to read and write Chinese and sign the informed consent form.

### Exclusion criteria

Subjects will be excluded if they meet any of the following criteria: (i) are unable to walk; (ii) have a serious infection of the knee; (iii) have suspected tears in any ligaments or menisci or acute inflammation of the synovial capsule; (iv) have a history of trauma, ligament damage, fracture, or surgery on the knee(s) within 6 months, causing pain or functional problems (history of knee replacement will be excluded); (v) have a history of local tumour/malignancy at the knee; (vi) have physical or laboratory findings indicating infection, presence of autoimmune disease or inflammatory arthritis; (vii) have knee pain caused by radiculopathy/herniation of an intervertebral disc; (viii) have end-stage diseases or other suspected severe conditions such as deep vein thrombosis of the lower limb, oedema related to cancer or cancer treatment, severe blood coagulation disorders, uncontrolled systemic arterial hypertension and severe diabetes; (ix) have a history of prolotherapy, hyaluronic acid injections or corticosteroids injections within 3 months; (x) have received acupuncture, electro-acupuncture, Tui-na therapy, massage, or physiotherapy 8 weeks prior to enrolment in the trial; (xi) have severe pain in other regions; (xii) have severe mental disorder(s); (xiii) are oversensitive to needles; and (xiv) are insensitive to pain due to advanced diabetes, neuropathy or use of strong painkillers.

Eligible subjects will receive baseline assessments and will be randomly allocated to the sinew acupuncture group (*n* = 43) or the sham acupuncture group (*n* = 43) in a ratio of 1:1 by block randomization. The random digits and letters will be generated by SPSS, covered by aluminium foil, and sealed into opaque envelopes.

Subjects will be advised not to receive other acupuncture treatments, Tui-na, massage or physiotherapy. Subjects are not restricted from the use of painkillers (including herbs) or external ointments if they suffer intolerable pain. The use of rescue medications, the daily level of morning stiffness and exercise time will be recorded in patient diaries.

### Interventions

#### Sinew acupuncture group

Subjects receiving sinew acupuncture will sit on a chair with the knee joint flexed at a most comfortable angle as close to 90 degrees as possible (if required, a block of A4 papers will be placed under the foot to adjust the angle). A hospital trolley table will be set up to block the vision of the subject to his/her knee (for blinding purposes). Routine disinfection will then be performed by the acupuncturist with a 75% alcohol pad. Acupoints (1–2 cm away from the point of tenderness, spasm or pain) along the meridian sinews near the knee(s) will be punctured through the skin by using sterile needles with a size of 0.30 mm × 40 mm (MOCM®) at an angle of 0–10 degrees pointing along the direction of the pain and the meridian sinew. The needles will be withdrawn immediately to a depth just under the skin and inserted forward smoothly 10 mm to 20 mm to avoid inducing pain. Five to eight points will be selected on each painful knee (while walking and climbing stairs) based on the theory of sinew acupuncture. Adjustments of the needles will be performed by extension and flexion of the knee joint to ensure that the needles do not cause pain during movement. Needles will be covered immediately by hypoallergenic bandages. The trolley table will be pulled away. Subjects will be advised to walk for 10 min (including the needle adjustment time). Needles will be adjusted at any time during walking if the subject has any pain induced by the acupuncture needle (for safety purposes) or primary pain of KOA (for the purpose of enhancing treatment effect). The trolley table will be used for blinding when the acupuncturist adjusts the needles. The acupuncturist will record the details of every adjustment procedure. After 10 min of walking, patients will be advised to step up and down from a step (~ 18 cm in height) for 12 rounds per knee. The pain points will be examined, and needle adjustments will be performed if necessary, followed by a period of rest by sitting for 5 min. After treatment, the needles will be removed while the subject is in a sitting position with the trolley table in place for blinding. Bandages will be applied in the same position for continuous blinding. The entire procedure will last 30 min per session. Subjects will receive 10 sessions of interventions for 4 weeks, starting with thrice a week during the first 2 weeks and then twice a week during the last 2 weeks. The above procedures will be performed by the acupuncturist and blinded to subjects.

#### Sham acupuncture group

Subjects in the sham acupuncture group will undergo the same procedures as those in the sinew acupuncture group except that the non-insertion sham acupuncture will be applied. Briefly, subjects will sit in a chair and their vision of their knees will be blocked by the trolley table. After disinfection, sterile needles with a size of 0.30 mm × 40 mm (MOCM ®) will be used to slightly puncture the acupoint without passing through the skin. The needles will immediately be covered with non-allergenic bandages to ensure sufficient blinding.

### Outcome measures

The VAS at week 4 will be the primary outcome. The WOMAC, TUG, 8-step SCT, and SF-36 scores will be the secondary outcomes. The assessment schedule is shown in Table 1. Independent assessors who are not involved in acupuncture treatments will perform the VAS assessments (asking subjects to indicate the pain intensity at the most painful points during walking and ascending/descending a step and overall). Acupuncturists will complete a “Physical Examination and Treatment Form” including details of the medical history, diagnosis and treatment procedures. The acupuncturists will communicate with the subjects using neutral language. The administration of analgesics, external ointments and herbal medicines and the time of daily exercises will be collected weekly from patient diaries during the intervention period and at each visit during the follow-up period.

**Table 1:** The schedule of enrollment, interventions, and assessments

Items	Enrollment	Allocation (baseline)	Interventions (± 2 days)										Follow-up	
Time points (week)	-2	0	1			2			3		4		6	10
Treatment sessions (n)	–	–	1	2	3	4	5	6	7	8	9	10	–	–
Eligibility screen	✓													
Informed consent	✓													
VAS		✓	✓	✓	✓	✓	✓	✓	✓	✓	✓	✓	✓	✓
WOMAC		✓			✓			✓		✓		✓	✓	✓
SF-36		✓										✓		✓
TUG		✓						✓				✓	✓	✓
8-step SCT		✓						✓				✓	✓	✓
PE & Tx		✓	✓	✓	✓	✓	✓	✓	✓	✓	✓	✓	✓	✓
Patient Diary					✓			✓		✓		✓	✓	✓
Credibility Test								✓				✓		
Test of Blinding Success												✓		
Adverse events			✓	✓	✓	✓	✓	✓	✓	✓	✓	✓	✓	✓

PE & Tx, Physical Examination and Treatment Form; SCT, stair climb test; SF-36, 36-item short form; TUG, timed up & go test; VAS, visual analog scale; WOMAC, Western Ontario and McMasters University Osteoarthritis Index

### Measurement instruments

The 0–100 mm VAS is used to measure pain intensity. Subjects will indicate pain intensity on the VAS, with 0 mm representing no pain and 100 mm representing the most intolerable pain. Pain intensity will be measured at the most painful point during walking, the most painful point during ascending and descending a step, and the overall pain before and immediately after every treatment session and at follow-up at week 6 and week 10.

The WOMAC (Hong Kong Cantonese Version) [10] will be completed by subjects and will assess three domains: pain (5 items), stiffness (2 items) and physical function (17 items). The WOMAC will be measured at baseline, every week for the first 4 weeks, week 6 and week 10.

The SF-36 (Hong Kong Cantonese version) will be used to assess general QOL. The QOL will be collected by assessors at baseline, week 4 and week 10.

TUG and 8-step SCT will be evaluated by assessors at baseline, every 2 weeks after the last treatment session, and at week 6 and week 10.

### Assessment of credibility

The Credibility of Treatment Rating Scale will be used to assess the credibility of the acupuncture treatments after the 6th treatment at week 2 and the last treatment at week 4. The 4-item scale is specifically designed to assess the credibility of acupuncture. The following questions will be asked to each subject by the assessors: (1) Do you believe this treatment will reduce the pain you are suffering? (2) Would you recommend this treatment to a friend or relative with the same problem? (3) Does the treatment seem to be a logical one? (4) Do you believe this treatment could be very effective in curing your knee osteoarthritis? [30].

### Assessment of blinding success for acupuncture treatment

The blinding success of subjects will be evaluated by assessors after the last treatment session at week 4. Subjects in both groups will be asked the following question: “When you volunteered for the trial, you were informed that you had an equal chance of receiving sinew acupuncture or sham acupuncture treatment. Which acupuncture do you think you received?” Three options will be provided to the subjects, sinew acupuncture, sham acupuncture, and uncertain. Those who answered either sinew acupuncture or sham acupuncture will be asked to provide a reason for that assumption; the results will be recorded [12].

### Adverse events/serious adverse events

Subjects will be encouraged to report adverse events at each treatment session using the TESS-adverse event form. For serious adverse events such as death, life threatening events, significant or persistent disability/incapacity, hospitalization or prolongation of existing hospitalization, the date, time, vital signs and reasons for treatment assignment disclosure should be noted in the Severe Adverse Event (SAE) Form, reported to the Institutional Review Board (IRB) and monitored within 48 h. If there are medical concerns such that the treatment protocol has to be revised due to safety, sudden serious adverse events or ethical reasons, the revision must be approved by the IRB before implementation. If a subject withdraws from the study, the reasons for withdrawal will be recorded.

### Safety

Either sinew acupuncture or sham acupuncture will be performed by registered acupuncturists in Hong Kong who have at least 3 years of acupuncture experience. Throughout the trial, acupuncturists will follow the guideline from the Hong Kong Hospital Authority on Safety in Acupuncture for Chinese Medicine Practitioners (2nd amended version, 2016) including (1) prevention of fainting and syncope during acupuncture; (2) prevention of bleeding and bending or breaking of needles during acupuncture; and (3) prevention of performing acupuncture on the skin with local infections, ulcers, scars, or tumour. All acupuncture procedures adopted in our clinic (The Hong Kong Tuberculosis Association Chinese Medicine Clinic cum Training Centre of the University of Hong Kong) will follow (1) the introduction of the Notice for Patients Receiving Acupuncture and Tui-na Treatment at their first visit and (2) implementation of needle counting before and after each acupuncture session.

Adhesive bandages will consist of a flexible, non-woven, low-adherence contact layer and a low-allergy, acrylic adhesive backing layer, which will be used to maintain the needle position during movement.

### Potential risks and management

During the treatment, all acupoints will be located around the knee joint(s), and the needle insertion will be superficial/subcutaneous with standardized disinfection procedures. This method has very low risks of infection, pneumothorax or perforation of the viscera.

Small amounts of bleeding or bruising may occasionally occur. In clinical experience, a small amount of bleeding can be stopped by applying pressure with sterile cotton swabs/balls. Bruises usually disappear within 2–3 days.

A step test is used to evaluate the subjects’ pain points while ascending/descending steps. To avoid falls, the step will be placed near a treatment bed, and a clinical assistant will accompany the subject at all times. The stairwell on the same floor of the hospital equipped with handles, bright lighting and anti-slip coating will be used for SCT assessment. Assessors will accompany the subjects during TUG and SCT assessments.

### Ethics and dissemination

The ethical validity of the study has been assessed and approved by the Institutional Review Board of The University of Hong Kong/Hospital Authority Hong Kong West Cluster (HKU/HA HKW IRB, approved number UW 16–2007). The study was registered on ClinicalTrials.gov (Identifier: NCT03099317). The clinical study will be overseen by the HKU/HA HKW IRB. All subjects will receive sufficient information about the trial and must sign the informed consent form prior to enrolment. Case repot forms (CRFs) and study files will be archived for 3 years after completion of the final report and locked in a research cabinet. The Personal Data (Privacy) Ordinance (CAP 486) will be followed strictly.

### Sample size estimation

G-power was used for sample size determination. The sample size was estimated by the VAS value with sinew acupuncture for knee pain using data from our pilot study [29]. The study observed 85 sessions of acupuncture treatments on the knee. After acupuncture treatments, the average VAS was reduced from 38.9 to 21.0 (change = − 17.9) with a standard deviation = 10.7. We estimated that the sham acupuncture (17.9*60% = 10.74) could reduce 60% of pain intensity compared to true acupuncture. The effect size was 0.673. With α = 0.05 and power (1-β) =0.8, the sample size was determined to be 37 per group. With the consideration of a 15% dropout rate, 86 subjects are required in each of the two groups.

### Data analysis

All data will be doubly entered into a password-protected computer 1 week after data collection. The statistical analysis will be performed by SPSS 22.0 for Windows by a single statistician. The scores will be analysed by the intent-to-treat analysis. The last observation carried forward method will be used for missing data. Comparisons of continuous variables between the acupuncture and sham groups will be assessed using Student’s t-test or analysis of covariance (ANCOVA) with baseline measures as covariates. Comparisons of categorical data between groups will be tested by the χ^2^ test or the Mann–Whitney U-test. Changes in the scores from baseline within treatment groups will be assessed by the paired t-test or the Wilcoxon signed ranks test. Data from the patient diary will be assessed to analyse covariate balances in the two groups. If we find an imbalance, we will use the propensity score method [31] or the conditional inference method [32] to remove the confounding effect. All statistical tests will be two-sided at a 5% significance level.

## Discussion

KOA is one of the most common diseases among the elderly. This study will evaluate the efficacy of sinew acupuncture for pain reduction, functional movement improvement, and QOL improvement in KOA patients. In our clinic, there are 8–10 new KOA patients each month. We estimate that recruitment will be completed in 10 months.

The study is conducted in collaboration with the School of Chinese Medicine, Department of Diagnostic Radiology and Department of Orthopaedics & Traumatology, Li Ka Shing Faculty of Medicine, HKU. The eligibility of KOA subjects will be assessed by clinical physicians. The intervention will be performed by experienced acupuncturists. The subjects and assessors will be blinded to subjects’ allocations. The acupuncturist will not be involved in assessments or data entry. Data will be analysed by an independent statistician.

In acupuncture RCTs, it is critical to choose an appropriate control population. Vickers AJ et al. found that acupuncture was superior to both sham and no acupuncture controls for osteoarthritis pain with effect sizes of 0.16 and 0.57, respectively [18]. The small effect size in trials with sham acupuncture controls may be due to the following reasons: (1) the meta-analysis excluded outlying studies showing a very large effect size [18] and (2) according to Macpherson H, Vickers AJ, et al. in the same research group, acupuncture has a smaller effect size in trials with needle-insertion sham controls than in trials with needle non-insertion sham or non-sham controls [15]. Our previous study was consistent with their findings that acupuncture trials with needle non-insertion controls lead to more positive conclusions than those with needle-insertion sham controls [16]. In the present study, we will use needle non-insertion as the control, which may produce a smaller, nonspecific effect compared to needle-insertion sham controls. However, it should be noted that the effect size estimated from our pilot study [29] is larger than that of the previous study by Vickers AJ et al. [18]. If the power estimates prove to be high, the trail can be used to determine a more accurate effect size for future power calculations.

This trial will expand our knowledge of whether sinew acupuncture will reduce pain intensity, improve the symptoms and movements of KOA patients, and improve QOL. If this study is successful, the effectiveness of sinew acupuncture may be studied using a pragmatic trial design.

## Abbreviations

KOA: Knee osteoarthritis
QOL: Quality of life
SCT: Stair climb test
SF-36: 36-Item short form survey
TUG: Timed up & go test
VAS: Visual analogue scale
WOMAC: Western Ontario and McMasters University Osteoarthritis Index
